# Targeting the complexity of Src signalling in the tumour microenvironment of pancreatic cancer: from mechanism to therapy

**DOI:** 10.1111/febs.15011

**Published:** 2019-08-05

**Authors:** Ashleigh Parkin, Jennifer Man, Paul Timpson, Marina Pajic

**Affiliations:** ^1^ The Kinghorn Cancer Centre The Garvan Institute of Medical Research Sydney Australia; ^2^ Faculty of Medicine St Vincent's Clinical School University of NSW Sydney Australia

**Keywords:** focal adhesion kinase, integrin, microenvironment, pancreatic cancer, Src kinase, stroma

## Abstract

Pancreatic cancer, a disease with extremely poor prognosis, has been notoriously resistant to virtually all forms of treatment. The dynamic crosstalk that occurs between tumour cells and the surrounding stroma, frequently mediated by intricate Src/FAK signalling, is increasingly recognised as a key player in pancreatic tumourigenesis, disease progression and therapeutic resistance. These important cues are fundamental for defining the invasive potential of pancreatic tumours, and several components of the Src and downstream effector signalling have been proposed as potent anticancer therapeutic targets. Consequently, numerous agents that block this complex network are being extensively investigated as potential antiinvasive and antimetastatic therapeutic agents for this disease. In this review, we will discuss the latest evidence of Src signalling in PDAC progression, fibrotic response and resistance to therapy. We will examine future opportunities for the development and implementation of more effective combination regimens, targeting key components of the oncogenic Src signalling axis, and in the context of a precision medicine‐guided approach.

**Abbreviations**
Bcl2: B‐cell lymphoma 2
Cdk: cyclin‐dependent kinase
ECM: extracellular matrix
EGF: epidermal growth factor
EGFR: epidermal growth factor receptor
EMT: epithelial–mesenchymal transition
ERK: extracellular signal‐regulated kinase
ERK: extracellular signal‐regulated kinases
FAK: focal adhesion kinase
GLUT1: glucose transporter 1
GSK3beta: glycogen synthase kinase 3 beta
GTP: guanosine triphosphate
HA: hyaluronic acid
HGF: hepatocyte growth factor
HNSCC: head and neck squamous cell carcinoma
IL10: interleukin 10
IL6: interleukin 6
ITGA: integrin alpha‐3
JNK: Jun kinase
LAMA: laminin
MAPK: mitogen‐activated protein kinase
MAPK: mitogen‐activated protein kinases
MDM: minute 2 homolog
Mdm2: mouse double minute 2 homolog
MDSC: myeloid‐derived suppressor cell
MEK: mitogen‐activated protein kinase kinase
MMPs: metalloproteinases
mTOR: mammalian target of rapamycin
NF2: neurofibromin 2
NFkappaB: nuclear factor kappa‐light‐chain‐enhancer of activated B cells
PARP: poly‐ADP ribose polymerase
PD1: programmed cell death protein
PDAC: pancreatic ductal adenocarcinoma
PDGF: platelet‐derived growth factor
PIP: phosphatidylinositol 4,5‐bisphosphate
PTEN: phosphatase and tensin homolog
QCMG: Queensland Centre of Medical Genomics
Raf: rapidly accelerated fibrosarcoma
Rho: Ras homolog gene family
ROCK: Rho‐associated coiled‐coil containing protein kinase
TAM: tumour‐associated macrophage
TCGA: The Cancer Genome Atlas
TME: tumour microenvironment
TNF: Tumour necrosis factor
VEGF: vascular endothelial growth factor
VEGFR: vascular endothelial growth factor receptor
WGS: whole genome sequencing

## Introduction

Our definition of ‘cancer’ is constantly being revised, with the traditional definition of a malignancy derived from epithelial cells now being inapplicable 1. It is now well recognised that carcinomas are not simply collections of individual clonal tumour cells, but rather comprise a complex environment of distinct cell types including molecularly diverse malignant cells and supporting nontransformed components that promote cancer development, spread and therapeutic resistance 2. These include resident cancer‐associated fibroblasts, pericytes, endothelial cells, adipocytes, nerves and infiltrating immune cells, which through dynamic communication with tumour cells, collectively regulate tumour growth and progression 2.

Pancreatic ductal adenocarcinoma (PDAC) is a highly lethal malignancy with a dismal 5‐year survival of < 8%, and this statistic has remained largely unchanged for the past 50 years 3, 4. PDAC is the third leading cause of all cancer deaths and is predicted to become the second by 2030 3, representing a significant burden in the Western society 3, 4, 5. Combination of chemotherapy agents, fluorouracil [5‐FU], leucovorin, irinotecan and oxaliplatin (FOLFIRINOX) or gemcitabine and nanoparticle albumin‐bound paclitaxel (Abraxane) represent current first‐line treatments for advanced PDAC 6, 7, 8. As most recent data indicate, their efficacy may also be of significant benefit in both adjuvant 9 and neoadjuvant settings 10. However, due to the toxicity associated with multiagent chemotherapy, there is a discernible need for novel, more tailored treatment combinations, as well as the identification of biomarkers to help rationalise treatment selection 5.

PDAC has a high molecular heterogeneity despite being morphologically indistinguishable 11, 12. Characterisation of this complex molecular landscape has revealed key insights into the biology of tumours 11, 13, 14, enabling us to build upon the traditional anatomical definition of cancer and further includes molecular subtyping or ‘omic’ stratification as a foundation for developing approaches for early detection and improved treatment options 11, 15, 16, as well as identification of mechanisms of therapeutic resistance 11, 12, 17. With new advances in sequencing and analytical methodologies, PDAC has been genomically and transcriptomically characterised to an incredible depth, as reviewed recently 14. Building on early studies which have identified the 12 key pathways and oncogenes genetically altered in most pancreatic cancers 18, this disease has since been stratified into distinct molecular subtypes using gene expression profiling 17, and comprehensive whole genome sequencing (WGS) approaches 11, 12, 19. For example, these analyses have led to the identification of a PDAC subtype characterised by high structural variation (> 200 structural rearrangements per tumour), that may be preferentially sensitive to DNA‐damaging agents, including PARP inhibitors and cisplatin 11. Subsequent integrative analysis of genomic and transcriptomic signatures has further characterised an ‘immunogenic’ subtype in PDAC 12, associated with a significant immune infiltrate, with predominant expression profiles related to infiltrating B and T cells, upregulation of CTLA4 and PD1 immunosuppressive pathways, suggesting that a proportion of PDAC tumours may potentially be targeted with immune‐modulating agents. Further work by Connor *et al*. 19 has described an interesting correlation between signatures that define double‐stranded DNA break repair and mismatch repair deficiencies and specific immune profiles in pancreatic cancer, highlighting that similar to other solid cancers 20, a subset of pancreatic cancers with a high mutation burden may present a viable target for immune‐modulating combination therapies.

Moreover, comprehensive genomic and transcriptomic studies in more frequently occurring cancers, such as breast cancer, have not only transformed and improved our understanding of the tumour landscape, but have been utilised to refine breast cancer classification, assess prognosis and response to therapy 21, 22. These examples demonstrate how the identification of key mutations can clearly benefit a larger number of selected cancer patients, and illustrate the need to include a molecular taxonomy when establishing effective treatment plans.

In addition to the novel approaches to cancer treatment developed from the genomic characterisation of cancer cells within tumours, the equally complex and dynamic tumour microenvironment (TME) has been shown to play a significant role in promoting cancer development, progression and treatment failure. Of note, PDAC is characterised by a hypoxic, immunosuppressive and highly fibrotic environment, with stromal components outnumbering pancreatic cancer cells 23, 24. Intricate communication between pancreatic cancer cells and their surrounding environment, driven by a dynamic signalling network of cellular and matrix remodelling enzymes, cytokines, chemokines and growth factors, collectively promotes tumour growth and treatment resistance 25, 26, 27, 28.

A key pathway that regulates the tumour microenvironment is the Src signalling network. The c‐Src non‐receptor tyrosine kinase is frequently overexpressed in numerous human malignancies, including PDAC 29, where it has been shown to promote tumour development and progression to distant metastases, leading to poor patient survival. Moreover, Src kinase is a mediator of integrin signalling in pancreatic cancer cells 30, and plays an important role in the regulation of several proteins that are frequently deregulated in cancer including focal adhesion kinase (FAK), epidermal growth factor receptor (EGFR), Akt/PI 3‐kinase, and Rho/ROCK signalling. These pathways directly drive tumour‐cell to stromal‐cell crosstalk, 31, 32, 33, 34, 35 and play a prominent role in regulating pancreatic tumour cell survival, adhesion, migration and invasion 29. In this review, we summarise and discuss the current understanding of the diverse and complex roles of aberrant Src signalling in the complex niche of a rapidly developing and metastasising pancreatic tumour, highlighting challenges with and new avenues for the utilisation of inhibitors that target this dynamic network.

## The Src signalling axis promotes pancreatic cancer progression

**Figure 1:**
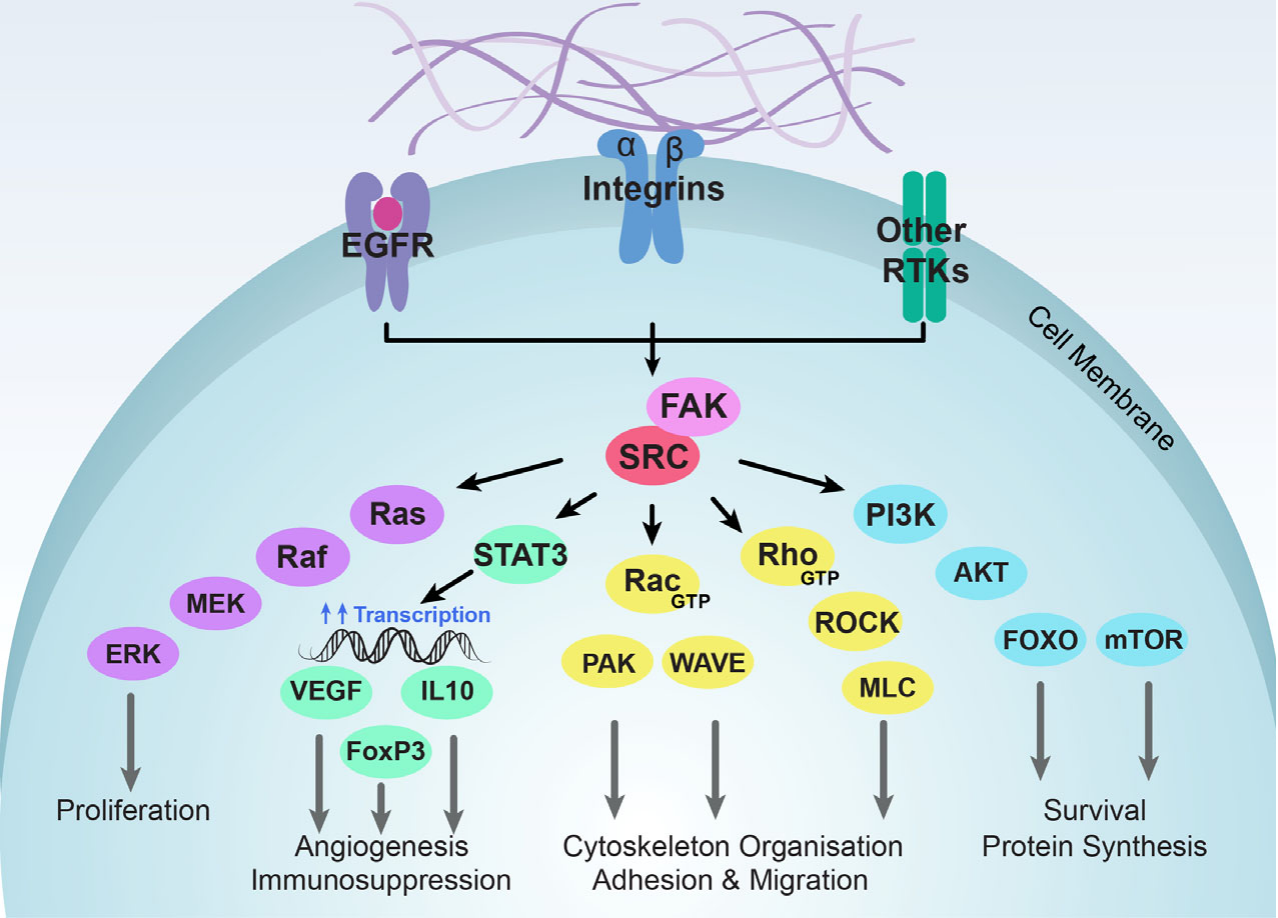
Schematic of the canonical Integrin/Src/FAK signalling network. Src and FAK interact with, and are activated by, numerous receptor tyrosine kinases (RTKs), including epidermal growth factor receptor (EGFR), vascular endothelial growth factor receptor (VEGFR), fibroblast growth factor receptor (FGFR), and platelet‐derived growth factor receptor (PDGFR), as well as the ‘matrix receptor’ integrins, which all facilitate their downstream signalling. (a) Phosphorylation and activation of RAS, RAF, MEK1/2 and ERK1/2 leads to the transcriptional regulation of genes associated with cell growth and proliferation. (b) Phosphorylation of signal transducer and activator of transcription 3 (STAT3), enables STAT3 dimerisation and translocation into the nucleus where it regulates gene expression of VEGF, IL10 and FoxP3, stimulating angiogenesis and immunosuppression. (c) PI3K assists in the recruitment of Akt to the plasma membrane, where it is phosphorylated and activated by PDK1/2, and then translocates to the cytosol or nucleus. Through its downstream mediators, Akt promotes RNA translation and protein synthesis, and cell survival. (d) Activation of Rho GTPases results in the binding of Rho‐associated protein kinase (ROCK) leading to actin cytoskeleton remodelling and cell motility. Rho GTPases can also activate myosin‐light chain (MLC) which is involved in the maintenance of stromal feedback and extracellular matrix deposition. Activation of Rac GTPases leads to the recruitment and activation of Arp2/3 via WAVE, leading to the formation of new actin polymers, whilst Rac can also activate PAK, leading to the inhibition of depolarisation of actin, key processes affecting actin dynamics and lamellipodia formation.

The proto‐oncogene tyrosine‐protein kinase Src or cellular Src (c‐Src) belongs to a family of nine nonreceptor tyrosine kinases that share similar structure and function 36. Src kinase localises at cell–matrix adhesions, and is readily activated by positive migratory growth factor signalling, including, but not limited to, epidermal growth factor (EGF), hepatocyte growth factor (HGF), platelet‐derived growth factor (PDGF), vascular endothelial growth factor (VEGF) and integrin 37 and Eph receptor (EphA2) activation 38. In turn, Src can phosphorylate substrates from numerous molecular pathways and consequently promotes tumour cell survival, proliferation, cell adhesion, migration, invasion and angiogenesis, key hallmarks of cancer (Fig. 1) 29, 30, 39, 40, 41, 42, 43, 44. The roles of Src in tumourigenesis and metastasis are well established, with constitutive activation of Src being observed in a variety of cancers including breast, lung, colon, prostate and pancreas 29, 42, 45.

Src modulates integrin adhesions, cadherin‐mediated cell–cell adhesions and metalloproteinase expression, and it is this disruption of intercellular adhesion that results in the detachment of tumour cells from the tumour mass, allowing them to invade through the extracellular matrix (ECM), penetrate the blood vessels and metastasize to other sites 43. Furthermore, Src kinase activity is required for mesenchymal invasion (involving integrin and protease‐dependent stromal remodelling) as it controls the turnover of integrin‐based adhesions 46. In addition, Src has been suggested as a mechanistic link between inflammation and cancer 47. Specifically, Src activation in tumour‐associated macrophages, leads to their increased motility and infiltration into the tumour, a process which is driven by the secretion of pro‐inflammatory cytokines within the tumour microenvironment 47, 48, 49. Src also plays a role in the metabolic reprogramming of cancers by promoting the Warburg effect. This involves activation of hexokinases and upregulation of glycolysis, which in turn promotes tumourigenesis 45.

The significance of Src in PDAC tumourigenesis is also well established 29, 48, 50. Src kinase expression and activity is upregulated in PDAC, increased further during progression to invasive and metastatic (advanced) PDAC and is associated with poor survival 29, 50, 51. Src also plays a role in the progression of pancreatitis, an inflammatory condition that presents a risk for development of pancreatic cancer 52. Similar to other cancers, Src inhibition has been shown to reduce proliferation, migration and invasion in PDAC cell lines*,* as well as inhibits tumour progression and metastasis *in vivo* 43, 53, 54, 55, 56, 57. Src can also promote the progression of PDAC by reducing tumour response to gemcitabine, one of the current standards of care chemotherapies for this cancer 58.

In addition to SRC, the integrin–focal adhesion signalling‐mediated modulation of ECM mechanics and cytoskeleton stability involves several important sensor proteins that are also frequently deregulated in cancer, including integrins, FAK and downstream Akt/PI 3‐kinase, LIM kinase, and Rho/ROCK activation 59, 60, 61, 62 (Fig. 1). Integrins are composed of two noncovalently associated transmembrane glycoprotein subunits, and can be divided into several subtypes 63. These molecules can signal bidirectionally: through the recruitment of adaptor proteins the integrin receptor becomes activated and has a high affinity for ECM ligands, which in turn leads to the recruitment of signalling proteins and the assembly of focal adhesions 63. Integrins bind to, and remodel ECM components such as vitronectin, laminin, fibronectin and collagen, thereby providing the traction required for tumour cell motility and invasion. Increased deposition and cross‐linking of ECM proteins can also further promote tumour progression via mechanical force‐induced clustering of integrin receptors 64.

The crosstalk between integrins, growth factor receptors and SRC oncogene is readily exploited by cancer cells during both tumour initiation and disease progression 59. Furthermore, integrins also play a role in angiogenesis, by providing a docking site for several cell types, including endothelial cells, endothelial stem cells and inflammatory cells, at the site of angiogenesis 65. Upregulation of ανβ6‐integrins occurs in a variety of tumours, including PDAC, where it has been shown to activate TGF‐β, stimulating tumour cell epithelial‐to‐mesenchymal transition (EMT) and stromal myofibroblast differentiation 66, which has in turn been shown to either promote 67 or restrict tumour growth and progression 68. The association between ανβ6‐integrins and increased migration, invasion and cell survival is partly due to the regulation of proteases (MMPs), and urokinase‐type plasminogen activator (uPA) 63, 66, 69, 70, 71. In PDAC specifically, overexpression of integrin ανβ3/ανβ6 has been previously shown to associate with poor survival of patients as well as lymph node metastasis 59, 72, and recent findings indicate that the stromal localisation and levels of active α5β1‐integrin and FAK can identify two readily distinguishable desmoplastic phenotypes in pancreatic cancer. Tumours with high stromal pSMAD2/3 levels were found to be prognostic of poor outcome, whilst increased stromal levels of active α,β‐integrin constituted a patient‐protective PDAC‐associated desmoplastic phenotype 73. In addition, integrins also play a role in regulating cancer stem cell properties leading to metastasis as well as resistance to tyrosine kinase inhibitors in PDAC 74.

Focal adhesion kinase (FAK) is a ubiquitously expressed nonreceptor tyrosine kinase that regulates integrin‐mediated cell‐ECM signalling, and its phosphorylation and activation is dependent on Src. The Src‐FAK multiprotein complex localises at cell–matrix attachment sites and influences several downstream pathways including cell motility, migration, invasion, survival, immunosuppression and apoptosis 25, 29, 75, 76. The mechanisms involved are complex but often include the regulation of downstream effectors, including TGFβ, as well as regulators of ERK, Jun kinase (JNK) and Rho signalling pathways 34, 35, 42, 77, 78, 79. FAK is overexpressed in a variety of cancers including PDAC, and overexpression is associated with poor prognosis 76, 80. It has recently been shown that FAK plays an important role in regulating pro‐inflammatory pathway activation and cytokine production during wound healing 25, 44, 80, 81, 82, 83. In PDAC specifically, FAK activity has been shown to correlate with high levels of fibrosis and poor CD8+ cytotoxic T‐cell infiltration, making it a promising target to overcome the highly fibrotic and immunosuppressive nature of PDAC 25, 84.

Src‐family kinases (SFKs) not only promote cell–matrix adhesion turnover through FAK, but also regulate Rho family of small GTPases, in particular RhoA and Rac1 activation 85, 86. Rho GTPases are often hijacked by cancers because they regulate diverse cellular processes that are important for tumour growth and metastasis including cytoskeletal dynamics, motility, contractility, cell polarity, membrane transport, gene transcription, as well as regulating the interaction between stromal cells and cancer cells 87, 88, 89, 90, 91, 92, 93. SFKs control the regulatory molecules of Rho GTPases (guanine nucleotide exchange factors (GEFs), GTPase‐activating proteins (GAPs) and guanine dissociation inhibitors (GDIs)), and it is the tight regulation and extensive crosstalk between Src/FAK and Src/RhoA/Rac1 that controls integrin‐mediated cell adhesion and migration 94, 95, 96. We have recently reviewed the role of Rho‐associated kinase signalling in cancers including PDAC 87, 88.

PI 3‐kinase (PI3K) signalling is another relevant, tumour‐promoting and potentially druggable effector network activated through FAK/SFK 97, 98, 99. Activated PI3K phosphorylates phosphatidylinositol 4,5‐biphosphate (PIP_2_) to produce PIP_3_, and this process is negatively regulated by PTEN 100. Activation of PIP_3_ can then further activate Akt (Akt activation occurs in ~ 59% PDAC samples 101) and additional downstream targets such as Bcl‐2, Mdm2, GSK3beta, NF‐kappaB and mTOR 97, 102, ultimately promoting cancer cell survival, growth, and motility and inhibiting apoptosis 97, 100, 103, 104. The PI3K‐Akt‐mTOR pathway is also responsible for controlling cellular metabolism. Oncogenic K‐Ras can enhance the activity of the metabolic enzyme ATP citrate lyase in an Akt‐dependent manner leading to histone acetylation and alteration of the acetyl‐CoA pool, subsequently leading to changes in gene expression, DNA damage response and DNA replication 105. The PI3K/Akt pathway can also inhibit glucose metabolism by blocking glycogen synthase kinase 3β and can alter glucose uptake by mediating expression of glucose transporters such as GLUT1 105, 106. Furthermore, Akt signalling is present in preneoplastic lesions during pancreatic carcinogenesis induced by mutated Kras, and is associated with progression towards higher grade tumours and poorer patient survival 99, 107, 108, 109.

## Molecular and genomic aberrations of the Src signalling axis in Pancreatic Cancer: Implications for therapeutic targeting

**Figure 2:**
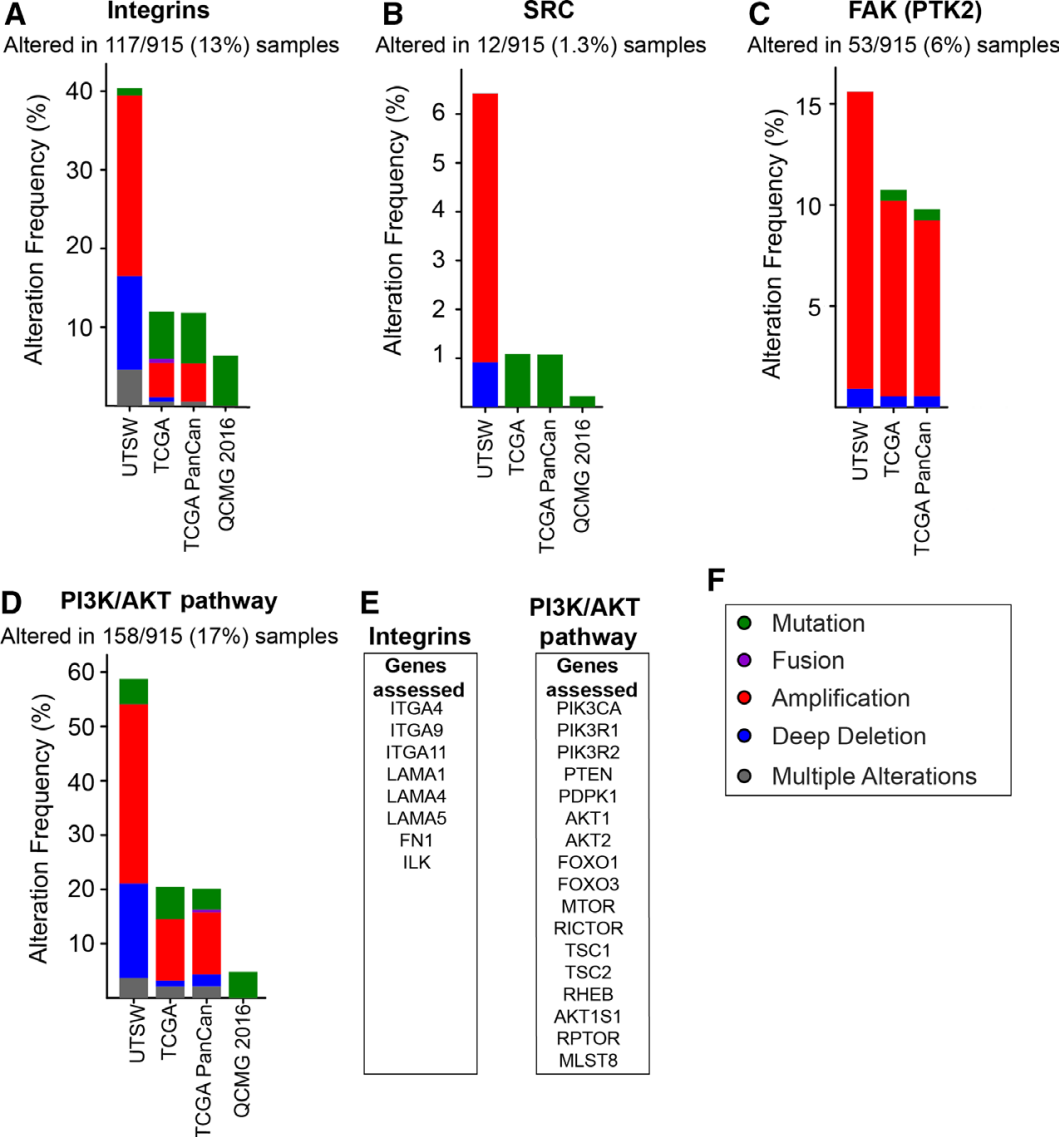
Genetic alteration frequency (% of patients) for key Src signalling components, generated from publically available pancreatic cancer genomics datasets. These datasets include The Cancer Genome Atlas (TCGA), PanCan Atlas (TCGA PanCan), University of Texas South Western Medical Centre (UTSW) and Queensland Centre for Medical Genomics (QCMG 2016) cohorts 114, 115. The genetic alterations examined include mutations (green), fusions (purple), amplifications (red), deletions (blue) and multiple alterations (grey) (F). (A) Genetic alteration frequency of integrins, with integrin genes being defined in (E). (B) Genetic alteration frequency of Src. (C) Genetic alteration frequency of FAK (PTK2). (D) Genetic alteration frequency of the PI3K/AKT pathway. (E) The list of genes used to define the PI3K/AKT pathway. Figure reproduced from Refs 114, 115

Historically, the documented cases of activating Src mutations are rare, with only one major study in colon cancer documenting 12% of cases with a truncating mutation at codon 531 110, which when functionally validated, was shown to lead to increased Src specificity and transformation of NIH 3T3 cells. Despite this, other studies using larger colon cancer populations document no such mutations 111, 112. In addition, no such mutations have been documented for Src‐implicated cancers, such as haematological malignancies 113. In PDAC specifically, examination of multidimensional publically available cancer genomics datasets (TCGA, PanCan Atlas and QCMG cohorts) revealed that Src mutations occur at a frequency of less than 2% (Fig. 2B) 114, 115, indicating that aberrant intratumoural Src activity occurs through constitutive activation of Src, or by changes in the levels of regulators of Src and amplification of downstream signalling pathways 113, 116, 117, 118.

Integrins are key regulators of Src signalling, and are also deregulated in cancers, but are rarely mutated. Several cancers, including glioblastoma, show modifications of the integrin pattern to be associated with tumour progression and poor patient survival, including α6β4, α6β1, αvβ6 and αvβ3 119. An early sequencing study demonstrated a positive association between mutations in subunit α7 (encoded by *ITGA7* gene), identified in 57% of prostate cancers, and increased cancer recurrence 120. The mutation also occurred in 21% of hepatocellular carcinomas and 83% of glioblastomas, as well as leiomyosarcomas 120. Decreased integrin expression has also been correlated with cancer progression. In mesothelioma, reduced expression of ITGA7 was associated with promoter methylation and was identified as an important mechanism for the aggressive migratory transformation of mesothelioma 121, 122. Similar results have also been seen with α2β1 in breast cancer, and α6β4/ α6β1 in oesophageal carcinoma 59. In PDAC, early sequencing studies identified genetic alterations in the integrin signalling pathway (*ITGA4*,* ITGA9*,* ITGA11*,* LAMA1*,* LAMA4*,* LAMA5*,* FN1* and *ILK*) in 67% of tumours 18. However, these alterations appear less frequent (67% versus 13%) when compared to the findings of the TCGA, UTSW, ICGC and QCMG 114, 115, 123 (Fig. 2A). This inconsistency may be explained through the study design of Jones *et al*. 124, where only small cohorts derived from cell lines (commercial and patient‐derived; *n *= 24); and xenograft models (*n *= 90) were used to analyse the mutational cancer landscape. Recent findings suggest that molecular landscapes of patient‐derived models may diverge from their parental tumours during long‐term propagation. More recently, the integrin β4 subunit was found to be commonly overexpressed in PDAC and is an adverse prognostic marker; however, it is not commonly mutated 125. An alternate mechanism involving a mutation in *TP53* is thought to promote integrin α6β4‐mediated tumour cell survival 125.

In addition, recent large‐scale, pan‐cancer proteogenomic studies have identified molecular alterations in several Src effector networks including PI3K/Akt/mTOR and FAK 80, 126, 127, 128. Of > 7000 tumours examined, 63% harboured nonsilent somatic mutations or copy number alterations within the PI3K/AKT/mTOR pathway 127. In PDAC specifically, ~ 17% of tumours carried alterations, the majority of which involved gene amplification, and this finding is consistent across multiple cohorts 114, 115 (Fig. 2D). The *PI3KCA* gene mutations present in 3–5% of pancreatic cancer patients can act as activating mutations initiating pancreatic tumour formation 129. Further, inactivating aberrations in PTEN (negative regulator of PI3K/PI3K pathway) occur in up to 70% of human PDAC, and have been shown to activate the tumour‐promoting stromal and immune cell components that shape the PDAC TME 130. FAK is also frequently overexpressed and deregulated in PDAC, with genomics alterations occurring at a frequency of ~ 6%, the majority of which are gene amplifications (Fig. 2C) 114, 115. FAK inhibitor monotherapy has shown mixed clinical efficacy in mesothelioma tumours that harbour loss of specific tumour suppressive signals, such as Merlin (encoded by *NF*2 gene; 131, 132, 133). Although mutations at the *NF*2 locus are rare (~ 10%) in human PDAC 12, 19, Merlin expression is lost in > 40% of PDAC and is negatively correlated with tumour stage, regional lymph node metastasis and differentiation 134. Assessment into the efficacy of FAK inhibition in the context of Merlin loss, and combined with additional biomarkers, in PDAC may be of interest.

A personalised treatment strategy using pharmacological inhibition of Src, Src‐associated regulators or downstream targets, in tumour subtypes carrying these aberrations, could be beneficial and remains to be examined. Currently there are no FDA‐approved prognostic or predictive biomarkers for PDAC 7. Importantly, moving forward, the integration of DNA copy‐number alterations, methylome, mRNA and protein, metabolomics and clinical information may help to further delineate the extent of Src signalling deregulation in pancreatic and other cancers, and could potentially lay the foundation for more accurate and rapid implementation of therapeutic inhibitors of Src as personalised cancer therapeutics.

## Targeting Src kinase in pancreatic cancer

Recognising the established role of Src in cancer initiation and progression led to the rapid development of several small molecule inhibitors (Table 1) 135. Inhibitors including bosutinib, saracatinib and dasatinib have shown measurable antitumour activity in several *in vitro* and *in vivo* models of cancer 47, 53, 56, 136, 137, 138. Dasatinib is a potent adenosine triphosphate‐competitive inhibitor of Src and Abl kinases, as well as c‐KIT, PDGFR and ephrin‐A2, which works by competitive inhibition of the ATP binding site. Its activity results in inhibition of cell proliferation (causing G_0_/G_1_ arrest), as well as inhibition of cell adhesion, migration, invasion and tumour metastasis 44, 53, 139, 140, 141, 142, 143. These results were particularly promising in models of advanced PDAC, presenting dasatinib as an encouraging antimetastatic agent for this disease 29, 56, 144. Despite the encouraging clinical results for the use of dasatinib as a standalone therapy in CML, clinical findings with dasatinib or alternative Src/ABL‐kinase inhibitors (saracatinib, bosutinib) 145, 146 in PDAC were predominately negative, partially due to poor drug tolerance, but also due to the highly aggressive and adaptable nature of this disease to single‐agent targeted therapies and rapid onset of resistance 53, 138, 147, 148, 149, 150, 151, 152, 153, 154, 155, 156. Moreover, the presumption that these biologic agents would significantly improve survival in nonstratified cohorts, particularly in PDAC, is inconsistent with prior preclinical data, which suggests that therapeutic response may correlate with biological markers. For example, Saracatinib effectively inhibited the growth of three patient‐derived pancreatic xenografts characterised by decreased FAK, paxillin and STAT3 signalling 136. In addition Bosutinib sensitivity was shown to correlate with caveolin 1 expression 138, and clinical trial data indicate that selected individuals experienced durable and sustained responses to dasatinib treatment 102, 150, 151. Collectively, these data highlight the need for further investigation into the biological ‘omics’ of patients prior to treatment in order to identify the mechanistic rationale that can predict which patients may most optimally respond to Src‐based therapies.

**Table 1:** Clinical trials in pancreatic cancer associated with targeting Src kinase.

Signalling pathway	Agent	Molecular target	Cancer type	Phase	Combination therapy	Findings/status	Protocol ID	Reference
Src	Dasatinib	Src, Abl, PDGFR	Metastatic pancreatic cancer	II (single arm)	Monotherapy	Completed: no significant clinical activity measured (*n *= 34); 1 durable sustained response on therapy (> 20 months), plus 6 long‐term survivors noted (> 20 months)	NCT00474812	150
			Metastatic pancreatic cancer	II (single arm)	Monotherapy	Terminated: Due to toxicity (*n *= 7)	NCT00544908	
			Molecular analysis for therapy choice (MATCH), multiple solid cancers incl metastatic or recurrent pancreatic cancer	II (personalised)	Monotherapy‐targeted against *DDR*2 mutations	Recruiting	NCT02465060	
			Metastatic pancreatic cancer	I	Gemcitabine	Terminated: Due to low accrual	NCT00598091	
			Locally advanced pancreatic cancer	II (randomised)	Gemcitabine	Completed: no significant improvement in PFS, OS in unselected patient cohort (*n *= 202). High dose regimen utilised leading to significant adverse events	NCT01395017	155
			Resected pancreatic cancer (adjuvant)	II (randomised)	Gemcitabine	Completed: awaiting results	NCT01234935	
			Advanced pancreatic cancer	I	Erlotinib + gemcitabine	Active, not recruiting. Well tolerated. Early clinical activity with reported OS 8 months and disease control rate 69% vs historical control OS 5.9 months and 58% respectively. Small patient cohort (*n *= 19)	NCT01660971	185
			Metastatic pancreatic cancer	II (single arm)	mFOLFOX6	Active, not recruiting (*n *= 38)	NCT01652976	137
	Bosutinib	Src, Abl	Advanced solid cancers (incl pancreatic)	I	Monotherapy	Completed: MTD determined; no significant efficacy observed	NCT00195260	154
			Resected pancreatic cancer	I	Gemcitabine	Terminated: Due to slow accrual	NCT01025570	
			Locally advanced/metastatic solid cancers (incl pancreatic)	I/II	Capecitabine	Terminated: Tolerated, limited efficacy overall (*n *= 5 pancreatic cancer patients)	NCT00959946	156
	Saracatinib (AZD0530)	Src	Recurrent metastatic pancreatic cancer	II (single arm)	Monotherapy	Completed: no objective response observed in unselected cohort (*n *= 19)	NCT00735917	138
			Advanced pancreatic cancer	I/II (Single Arm)	Gemcitabine	Completed: well tolerated but no improvement in efficacy over Gemcitabine alone	NCT00265876	153
			Advanced solid cancers (incl pancreatic)	I	Cediranib (VEGFR1 inhibitor)	Completed: tolerated. Demonstrated stable disease as best response in 22/35 evaluable patients	NCT00475956	256
	TNO155	SHP‐2	Advanced solid cancers	I	Monotherapy	Recruiting	NCT03114319	
	RMC‐4630		Advanced refractory solid cancers	I	Monotherapy	Recruiting	NCT03634982	

Given that in pancreatic (and other) cancers, multiple mechanisms often work in synchrony to lead to chemoresistance, considering more tailored treatment combinations that involve inhibition of Src, other molecular targets, plus tumour‐debulking cytotoxic agents may present a more effective approach. The rationale behind this includes the finding that Src is associated with increased chemoresistance in PDAC, and that inhibition of Src can overcome resistance to gemcitabine 58, 137, 143. Furthermore, Src inhibition is associated with decreased thymidylate synthase, which in turn is associated with the reversal of 5‐fluorouracil resistance 137. Src inhibition can also increase oxaliplatin activity, and inhibit oxaliplatin‐induced Src activation 137. When dasatinib was combined with gemcitabine in locally advanced pancreatic cancer, there was no improvement in progression‐free or overall survival (NCT01395017) (Table 1) 157. However, newer combination chemotherapy regimens, such as FOLFIRINOX 6, lead to significantly higher response rates and disease control in patients with metastatic disease. Hence, a potentially more appropriate future study design may involve sequential administration of dasatinib as ‘maintenance’ therapy, after optimal disease control is achieved with this highly active chemotherapy regimen (similar to successful previous studies utilising sunitinib 152), or alternatively a ‘priming regimen’ could be applied 92, thus limiting toxicity associated with chronic dosing.

The Src signalling network is also known to play an important role in the movement and infiltration of immune cells into the tumour. In addition Src activation is mediated by inflammatory cytokines within the tumour microenvironment, whilst also being involved in intercellular communication 47. Although there is minimal evidence in pancreatic cancer, research into other solid cancers including melanoma, sarcoma, colon and breast cancer demonstrates that Src‐inhibitors such as dasatinib have potent immunomodulatory functions 158, and consequently may present a promising adjunct to immunotherapy. Dasatinib may enhance cellular immunity through a number of mechanisms including T‐cell immunomodulation, whereby treatment has been shown to reduce the number of intratumoural regulatory T cells, in various solid tumour mouse models and haematological malignancies, promoting natural killer (NK) cell expansion and differentiation 158, 159, 160. In chronic myeloid leukaemia (CML) cancer models, dasatinib may increase the number of Granzyme B (GrB) expressing memory CD4+ T cells (GrB+CD4+ T‐cells) and promote their differentiation into Th1‐type T‐cells, which in turn produce interferon‐gamma, a powerful tumour‐suppressive cytokine 161. Moreover, in CML and head and neck cancers, dasatinib has been shown to reduce the number of myeloid‐derived suppressor cells (MDSCs), and induce anti‐inflammatory macrophages (defined by increased production of IL‐10, decreased production of IL6, IL‐12p40 and TNF‐alpha, and high expression of LIGHT, SPHK1 and arginase 1), via the inhibition of salt‐inducible kinases 160, 162, 163. Surprisingly, the potential in combining the immunomodulatory effects of Src‐inhibitors with other immunomodulatory therapies has not been extensively studied. Preclinical data in head and neck squamous cell carcinoma (HNSCC) showed inhibition of tumour growth, suggesting that combining dasatinib with anti‐CTLA4 immunotherapy may be a viable treatment approach 164. However in a clinical study of gastrointestinal stromal tumours (GIST), dasatinib and anti‐CTLA4 antibody ipilimumab were well tolerated yet the combination was not synergistic, potentially due to the lack of a biomarker‐driven approach 165. At present there is only one phase II trial underway examining the combination of dasatinib and anti‐PD‐1 therapy nivolumab in nonsmall cell lung cancer (NCT02750514). However due to the strong immunomodulatory effects of Src inhibition seen *in vivo*, assessment of synergistic combinatorial therapies including dasatinib and other immunomodulatory drugs is warranted. This could be particularly relevant in pancreatic cancer where immunotherapy provides no therapeutic benefit as a result of the immunosuppressive microenvironment that defines these tumours 166.

Combining Src inhibition with additional targeted therapies is another potentially beneficial approach aimed at enhancing antitumour efficacy, while minimising inherent and acquired resistance. This strategy has already shown promise in several cancers 167. Almost 30 years ago, Src tyrosine kinase and EGFR were found to synergistically stimulate EGF‐induced mitogenic cellular responses in fibroblast cultures 168. Since then, Src has been shown to directly phosphorylate EGFR and may also mediate transactivation of EGFR by other receptor signalling pathways 37, 169, 170. The EGF‐mediated RAS/RAF/MEK/ERK pathway (Fig. 1) is one of the major players in the regulation of tumour growth, survival, proliferation, inhibition of apoptosis and autophagy 171, 172, with deregulated activation associated with poor prognosis in solid tumours 173, including PDAC 174.

Targeting this key pro‐tumourigenic molecular pathway has been explored in PDAC with the combination of standard therapy gemcitabine and small molecule EGFR inhibitor erlotinib revealing a modest but significant improvement in patient survival in advanced disease 175, 176, 177. However, significance was lost when this combination was trialled in all‐comers in the adjuvant setting 178. Further analyses revealed that therapeutic benefit of combined gemcitabine/EGFR inhibition associated with KRAS wild‐type tumour status 179, 180 or development of skin rash in patients, which represents another measure of EGFR inhibitor activity 181. Dasatinib has been combined with the EGFR inhibitor, erlotinib in NSCLC, resulting in two partial responses, and a disease control rate of 63% 182. Collectively, these studies highlight the potential utility of this treatment combination when applied in small, but potentially well‐defined subgroups of patients with pancreatic cancer. Moreover, the combination of dasatinib, erlotinib and gemcitabine showed significant synergy in preclinical studies, with potent inhibition of cancer cell proliferation, viability and xenograft tumour growth 183. The triple combination was also shown to overcome constitutive activation of STAT3‐mediated signalling, a key player in PDAC chemoresistance 27, 55, 183, 184, and was shown to be well tolerated, with promising preliminary clinical activity in advanced pancreatic cancer 185. The potential of this therapeutic combination also provides support for the development of a novel multikinase inhibitor (SKLB261) that potently inhibits EGFR, Src and VEGFR2 kinases. In the context of PDAC, this inhibitor effectively inhibited cancer cell proliferation, migration, invasion and induced apoptosis *in vitro*, and demonstrated potent antiangiogenic effects in pancreatic cancer xenografts, with stronger antitumour activity when compared to dasatinib, erlotinib and gemcitabine monotherapies 186.

Dual Src/MEK blockade using saracatinib/selumetinib presents another interesting therapeutic strategy shown to induce apoptosis of dormant cancer cells and limit tumour recurrence in breast cancer models 187 that may potentially be applied to other solid cancers, including PDAC. Dual targeting of Src and the protein tyrosine phosphatase SHP‐2, required for full activation of the RAS/ERK1/2 pathway, has also shown promise in *in vitro* and *in vivo* models of pancreatic cancer. Combined Src/SHP‐2 inhibition resulted in a supra‐additive loss of phosphorylation of Akt and ERK‐1/2, and led to an increase in apoptotic marker expression in L3.6pl and PANC‐1 pancreatic cancer cells. The combination also led to a reduction in cell viability, adhesion, migration and invasion *in vitro* and reduction in pancreatic tumour formation *in vivo*, using the L3.6pl orthotopic model 188. The central role for SHP‐2 in oncogenic KRAS‐driven tumours has been therapeutically exploited in other contexts, with most recent data demonstrating potent synergistic antitumour effects of combined SHP‐2 and MEK inhibition in multiple cancer types 189, including genetically engineered models of KRAS‐mutant lung and pancreatic cancer 190. Further exploration of these targeted therapeutic combinations, particularly in molecularly enriched patient subsets, is warranted, with early dose‐finding clinical studies underway (NCT03114319, NCT03634982; Table 1).

## Modulation of the upstream and downstream Src signalling components in pancreatic cancer

Modulation of the downstream mediators and interacting partners of Src represents another potentially viable therapeutic approach that is increasingly being investigated (Table 2). Inhibition of FAK decreased PDAC cell growth and migration *in vitro* 191, 192, and limited pancreatic tumour progression *in vivo*, doubling the survival in the p48‐Cre;LSL‐KrasG12D;Trp53flox/+ (KPC) mouse model of PDAC 25, 193, 194. FAK inhibitor VS‐4718 treatment further reduced tumour fibrosis and numbers of infiltrating immunosuppressive populations of myeloid‐derived suppressor cells (MDSCs), tumour‐associated macrophages (TAMs) and regulatory T‐cells, sensitising the KPC mouse model to checkpoint immunotherapy 25. As a result, several trials are now focused on combining FAK inhibition with immunotherapies such as trametinib, and pembrolizumab in PDAC (NCT02428270 195, NCT02758587) (Table 2). In addition, FAK inhibitors such as PF‐00562271 are well tolerated and hence show significant promise for the treatment of PDAC 131, 196. Promising preclinical data in malignant pleural mesothelioma, ovarian and other solid tumours suggest that therapeutic responsiveness to FAK inhibition may be guided by Merlin loss 197, 198 or E‐cadherin levels 199. This is supported by positive data from two phase I studies (NCT01138033, NCT01938443) in advanced solid tumours, where improved response to the FAK inhibitor GSK2256098 was observed in Merlin‐negative mesothelioma 131, 133. However, findings of a recent prospective phase II trial in malignant pleural mesothelioma (MPM; COMMAND study), has since failed to confirm Merlin expression as a predictive biomarker of efficacy to a different FAK inhibitor, defactinib 132. The observed discordance in the findings of these studies could potentially be due to a substantial difference in the cut‐offs utilised to define Merlin‐negative or Merlin‐low tumour status, with the Soria *et al*. 131 and Mak *et al*. 133 trials more stringently defining Merlin‐negative cancers. These studies also differ in terms of their patient selection and cohort size, with the larger COMMAND trial 132 being a prospective study examining defactinib efficacy as a maintenance therapy in chemo‐responsive advanced MPM, whereas the smaller phase I and Ib studies of the GSK2256098 compound examined efficacy in advanced chemo‐resistant solid tumours, including mesothelioma. Moreover, as defactinib targets both FAK and Pyk2 200 while GSK2256098 is selective for FAK alone, this difference in target selectivity between the two compounds may potentially lead to divergent antitumour activity, and mechanism of action on tumour cells, as well as the distinct components of the tumour microenvironment. Further assessment into the efficacy of FAK inhibition in the context of Merlin loss may still be of interest, particularly in pancreatic cancer where it has yet to be examined. Future trials would however need to consider standardisation of the biomarker analysis and interpretation of Merlin loss, sampling of multiple tumour areas where possible to account for potential intratumoural heterogeneity of molecular marker(s) of interest and incorporation of additional promising biomarkers to aid identification of clinical responders to FAK inhibitor‐based treatment regimens.

**Table 2:** Clinical trials in pancreatic cancer associated with targeting downstream mediators and interacting partners of Src kinase.

Signalling pathway	Agent	Molecular target	Cancer type	Phase	Combination therapy	Findings/status	Protocol ID	Reference
EGFR	Erlotinib	EGFR	Advanced pancreatic cancer	III	Gemcitabine	Completed: modest significant improvement in OS (0.33 months) (*n *= 569). Association between rash and a better outcome was observed	NCT00026338	175
			Locally advanced pancreatic cancer	III	Gemcitabine	Completed: no significant improvement in OS in combination arm (1.7 months; *P* = 0.09; *n *= 449)	NCT00634725	257
			Advanced pancreatic cancer	II (Single Arm)	Gemcitabine	Completed: well tolerated, no significant improvement in PFS as primary measure in unselected cohort (*n *= 30)	NCT00810719	258
			Advanced pancreatic cancer	III	Cross‐over design (Gemcitabine vs Capecitabine)	Completed: well tolerated, comparable efficacy between the two Erlotinib‐based regimens (*n *= 274). KRAS wild‐type status was associated with an improved overall survival (HR 1.68, *P* = 0.005)	NCT00440167	176, 177
			Resected pancreatic cancer (adjuvant)	III (open label)	Gemcitabine	Completed: no improvement in patient survival observed (*n *= 436) and occurrence of rash was not associated with response	CONKO‐005	178
			Metastatic pancreatic cancer	II (single arm)	Gemcitabine	Completed: improved survival in rash‐positive patients, comparable 1% survival rate to FOLFIRINOX	NCT0172948	181
	Cetuximab	Chimeric monoclonal IgG_1_ antibody against extracellular III domain of EGFR	Advanced pancreatic cancer	III	Gemcitabine	Completed: no significant improvement in survival (*n *= 745) and no association with EGFR IHC	NCT00075686	259
	Nimotuzumab	Humanised IgG_2_ mAb against extracellular III domain of EGFR	Advanced pancreatic cancer	IIb (randomised)	Gemcitabine	Completed: safe and well tolerated. One‐year OS and PFS were significantly improved (*n *= 192). Particularly of benefit in KRAS wild‐type patients	NCT00561990	180
FAK	PF‐00562271	FAK	Advanced solid cancers (incl pancreatic)	I	Monotherapy	Completed: tolerated, MTD established. (*n *= 99; 14% pancreatic)	NCT00666926	196
	VS‐4718		Advanced pancreatic cancer	I	Gemcitabine/ Nab‐paclitaxel	Terminated: Company de‐prioritised drug development	NCT02651727	
	Defactinib		Molecular analysis for therapy choice (MATCH), multiple solid cancers (incl metastatic/ recurrent pancreatic cancer)	II (personalised)	Monotherapy‐targeted against *NF*2 inactivation	Recruiting	NCT02465060	
			Advanced solid cancers (incl pancreatic)	I/II	Pembrolizumab (anti‐PD1)	Recruiting	NCT02758587	
			Advanced solid cancers (incl pancreatic)	I	Pembrolizumab and Gemcitabine	Phase I Completed (*n *= 17). Well tolerated. Recruiting: Expansion cohort	NCT02546531	260
	GSK2256098		Recurrent pancreatic cancer	II (Single Arm)	Trametinib (MEK1/2 inhibitor)	Completed: no objective response measured in unselected cohort (*n *= 16). 1 patient with *KRAS* amplification showed stable disease for 5 months after rapid progression on First‐line FOLFIRINOX; Correlative biomarker studies ongoing from collected material	NCT02428270	195
Integrin	Cilengitide	Cyclic peptide inhibitor of ανβ3/ανβ5 integrins	Advanced pancreatic cancer	II (randomised, open label)	Gemcitabine	Completed: well tolerated, no improvements in OS, PFS and response rate in unselected cohort (*n *= 89)	EMD 121974	233
	Volociximab (M200)	Chimeric mAb against human α5β1 integrin	Metastatic pancreatic cancer	II (single arm, open label)	Gemcitabine	Completed: well tolerated, awaiting further results	NCT00401570	236
	IMGN388	Human IgG1 anti‐integrin Ab conjugated to maytansinoid (DM4)	Advanced solid cancers	I	Monotherapy	Completed: well tolerated, safety data reported on 26 patients; awaiting final results	NCT00721669	261
Hyaluronan	PEGPH20	Hyaluronan	Metastatic pancreatic cancer	Ib/II (randomised)	Gemcitabine	Completed: tolerated combination therapy, with promising early clinical activity, particularly in patients with HA‐high tumours (IHC). Phase II terminated due to change in standard‐of‐care chemotherapy treatment	NCT01453153	262
			Metastatic pancreatic cancer	II (randomised, open label)	Gemcitabine/ Nab‐paclitaxel	Completed: improved PFS as primary endpoint in the overall cohort (*n *= 279), with the greatest improvement in PFS observed in patients with HA‐high tumours (prevalence of 34%)	NCT01839487	248
			Advanced pancreatic cancer	NA (non‐randomised, open label)	Gemcitabine/ Nab‐paclitaxel	Recruiting: Interim results indicate adding Rivaroxaban is safe and effectively controls thromboembolic events, with PEGPH20‐combination therapy showing encouraging early responses (*n *= 28)	NCT02921022	252
			Borderline resectable pancreatic cancer (neoadjuvant)	II (single arm, open label)	Gemcitabine/ Nab‐paclitaxel	Recruiting	NCT02487277	263
			Metastatic pancreatic cancer	III (randomised)	Gemcitabine/ Nab‐paclitaxel	Recruiting	NCT02715804	
			Locally advanced pancreatic cancer	II (single arm, open label)	Gemcitabine and radiation	No longer recruiting, no results posted	NCT02910882	
			Metastatic pancreatic cancer	I/II	modified (m) FOLFIRINOX	Phase II closed as PEGPH20 with mFFOX caused significantly increased toxicity and decreased treatment duration compared to mFFOX alone	NCT01959139	253
			Resectable pancreatic cancer (neoadjuvant)	NA	Cetuximab	Study closed due to slow accrual	NCT02241187	264
			Advanced (chemotherapy‐resistant) pancreatic cancer	I	Avelumab	Recruiting	NCT03481920	
			Advanced (chemotherapy‐resistant) pancreatic cancer: HA high	II (single arm, open label)	Pembrolizumab	Not yet recruiting	NCT03634332	
			Metastatic pancreatic cancer	Ib/II (randomised, open label)	Atezolizumab	Recruiting	NCT03193190	
Rho/ROCK	AT13148	AGC Kinase	Advanced solid cancers	I	Monotherapy	Completed: tolerable, dose escalation ongoing (*n *= 30), awaiting final results	NCT01585701	201
PI3K/Akt Pathway	MK2206	Akt (pan)	Advanced pancreatic cancer	I/Ib (randomised, open label)	Dinaciclib (CDK inhibitor)	Completed: results pending	NCT01783171	
			Recurrent metastatic pancreatic cancer	II (randomised, open label)	Selumetinib (MEK1/2 inhibitor)	Completed:No improvement in OS, and increased rate of adverse events in experimental arm, compared to mFOLFOX standard therapy (*n *= 137)	NCT01658943	223
	Afuresertib (GSK2110183)	Akt (pan)	Advanced solid cancers (incl pancreatic)	I/II (open label)	Trametinib (MEK1/2 inhibitor)	Completed: Poor tolerability with daily dosing. Potential for intermittent administration discussed within study	NCT01476137	224
	Uprosertib (GSK2141795)	Akt (pan)	Advanced solid cancers (incl pancreatic)	I	Trametinib (MEK1/2 inhibitor)	Completed: results pending	NCT01138085	
	Oleandrin (PBI‐05204)	Akt (pan)	Metastatic pancreatic cancer	II (single arm, open label)	Monotherapy	Active, not recruiting	NCT02329717	
	AZD5363	Akt (pan)	Molecular analysis for therapy choice (MATCH), multiple solid cancers (incl metastatic/ recurrent pancreatic cancer)	II (personalised)	Monotherapy‐targeted against Akt mutations	Recruiting	NCT02465060	
	Perifosine	Akt (pan)	Advanced pancreatic cancer	II (single arm, open label)	Monotherapy	Completed: no results posted	NCT00053924	
			Advanced pancreatic cancer	II (single arm, open label)	Monotherapy	Terminated: Significant treatment‐related toxicity (*n *= 10). Disease progression noted	NCT00059982	265
	Alpelisib (BYL719)	PI3Kα	Advanced solid cancers (incl pancreatic neuroendocrine neoplasms)	Ib	Everolimus (mTOR) + Exemestane (Aromatase)	Active, not recruiting	NCT02077933	
			Advanced pancreatic cancer	I/Ib (single arm, open label)	Gemcitabine/ Nab‐paclitaxel	Active, not recruiting	NCT02155088	
	Buparlisib (BKM120)	PI3K (pan)	Metastatic pancreatic cancer	I (single arm, open label)	mFOLFOX6	Completed: results pending	NCT01571024	
			Advanced solid cancers (incl pancreatic)	Ib (single arm, open label)	Trametinib (MEK1/2 inhibitor)	Completed: long‐term tolerability of the combination was challenging, with promising efficacy in select tumour types (ovarian) (*n *= 113; 47 patients in the expansion cohort)	NCT01155453	222
			Advanced solid cancers (incl pancreatic)	Ib (single arm, open label)	MEK162 (MEK1/2 inhibitor)	Completed: results pending	NCT01363232	
	Sirolimus (Rapamycin)	mTORC1	Advanced (gemcitabine‐resistant) pancreatic cancer	II (single arm, open label)	Monotherapy	Completed: well tolerated, marginal efficacy, examined biomarker (p70S6K IHC) did not correlate with activity (*n *= 31)	NCT00499486	204
			Advanced pancreatic cancer	II (single arm, open label)	Monotherapy	Recruiting	NCT03662412	
			Advanced solid cancers (incl pancreatic ductal and acinar adenocarcinoma)	I	Vismodegib (Hedgehog inhibitor)	Suspended: results pending	NCT01537107	
			Advanced solid cancers	I	Sunitinib (RTK inhibitor)	Completed: results pending	NCT00583063	
			Advanced solid cancers	I	Sorafenib (Raf, VEGFR inhibitor)	Completed: results pending	NCT00449280	
			Metastatic pancreatic cancer	I/II (randomised, open label)	Metformin	Active, not recruiting	NCT02048384	
	SM‐88	Combination: metyrosine‐derivative + low‐dose sirolimus, phenytoin + methoxsalen	Metastatic (chemotherapy‐resistant) pancreatic cancer	II (randomised)	Monotherapy	Recruiting: Preliminary results are promising, with therapy well tolerated (*n *= 28), with a median of 4.3 months of follow‐up after treatment initiation, 67.8% still alive (trial ongoing), promising compared with historical data	NCT03512756	213
	Temsirolimus	mTORC1	Metastatic pancreatic cancer	II (single arm, open label)	Gemcitabine	Terminated	NCT00593008	
			Advanced solid cancers (incl pancreatic)	I/II (single arm, open label)	Nivolumab	Terminated: Investigator no longer at site to enrol patients or write up data	NCT02423954	
			Advanced pancreatic cancer	II (single arm, open label)	Monotherapy	Terminated: Study closed due to significant treatment‐related toxicity (*n *= 5). Disease progression noted in 2 patients	NCT00075647	266
	Everolimus (RAD001)	mTORC1	Advanced or metastatic pancreatic cancer	II (single arm, open label)	Erlotinib	Terminated: Study closed due to significant treatment‐related toxicity (*n *= 15). Lack of objective responses noted. Study suggests activation of negative feedback loops following mTOR inhibition may explain lack of efficacy, and which may require simultaneous inhibition of multiple PI3K pathway components to elicit response	NCT00640978	266
			Metastatic (gemcitabine‐resistant) pancreatic cancer	II (single arm, open label)	Monotherapy	Completed: well tolerated, minimal clinical activity as monotherapy in unselected cohort (*n *= 33)	NCT00409292	267
			Advanced or metastatic pancreatic cancer	I/II (randomised, open label)	Irinotecan and Cetuximab	Terminated: emergence of FOLFIRINOX and slow recruitment. Triple combination showed similar PFS but increased OS compared to Capecitabine + Oxaliplatin (7.7 vs 4.5 months *P *= 0.04) (*n *= 26)	NCT01042028	268
			Metastatic pancreatic cancer	II (non‐randomised, open label)	Capecitabine and Cetuximab	Completed: MTD determined; partial response documented in 2 patients (6.5%), and 5 (16.1%) had stable disease. Considerable epidermal and mucosal toxicities	NCT01077986	269
			Metastatic (gemcitabine refractory) pancreatic cancer	I/II (single arm, open label)	Sorafenib	Completed: awaiting results	NCT00981162	
			Advanced and/or metastatic pancreatic cancer	I/II (single arm, open label)	Gemcitabine	Completed: MTD determined. Clinical benefit (CR, PR or stable disease) observed in 78% patients (*n *= 21)	NCT00560963	270
			Pancreatic neuroendocrine tumours	I/II (open label)	X‐82 (VEGFR/PDGFR inhibitor)	Active, not recruiting. Prolonged stable disease (3‐23 months) (*n *= 10)	NCT01784861	271
			Advanced GI neuroendocrine tumours (incl pancreatic)	II (single arm, open label)	Monotherapy	Active, recruitment complete (*n *= 25). Early data indicate therapy is well tolerated with signs of efficacy (high rate of PR)	NCT01648465	272
	Vistusertib	mTORC1/2	Advanced solid cancers (incl pancreatic)	II (personalised, single arm)	Monotherapy‐targeted against RICTOR amplifications	Not yet recruiting	NCT03166904	
			Advanced solid cancers (incl pancreatic)	II (personalised, single arm)	Monotherapy‐targeted against TSC1/2 mutations	Not yet recruiting	NCT03166176	
	Dactolisib	PI3K/mTOR	Advanced solid cancers (incl pancreatic)	Ib (open label)	MEK162 (MEK1/2 inhibitor)	Completed: results pending	NCT01337765	
	Gedatolisib	PI3K/mTOR	Advanced solid cancers (incl pancreatic)	I (single arm, open label)	Palbociclib	Recruiting	NCT03065062	

Several inhibitors that target Rho GTPase or its downstream effectors including Rho‐associated kinases (ROCK) have shown antitumour activity in preclinical models, which we have reviewed previously 87, 88. Most recently, fasudil, an inexpensive, off‐patent ROCK inhibitor, may present a promising new treatment approach for PDAC. It has recently been shown that using a short‐term ‘priming’ treatment approach to inhibit ROCK signalling can reduce tissue stiffness, improve vascular patency, increase tumour perfusion, decrease *in vivo* primary tumour growth, metastasis and improve response to standard of care therapy 23, 92, similar to chronic fasudil treatment 89. Newer ROCK inhibitors (such as ripasudil, CCT129254 or AT13148), are currently being trialled, and utilise a similar ‘priming’ 92, 93 or intermittent regime 201. The rationale behind this novel treatment scheduling involves modulating or ‘loosening’ the ECM, via ROCK inhibition, prior to chemotherapy administration in order to improve chemotherapy drug perfusion and reduce toxicity 92. Potentially, this regime could be applied for the use of other stromal‐based therapies in PDAC as well as other stromal‐driven cancers.

Furthermore, there has been significant research dedicated to targeting the PI3K/AKT signalling pathway in PDAC due to its role in cell metabolism, cell cycle, protein synthesis and apoptosis 202. Rapamycin, an mTORC1 inhibitor, showed promising preclinical results in PDAC, significantly halting disease progression in PI3K/AKT‐activated tumours 203. However clinical data failed to demonstrate a benefit, particularly when administered as monotherapy (Table 2) 204. This may further be explained by mTORC1 being involved in complex negative feedback loops that restrain upstream signalling. For example, inhibition of mTORC1 drives activation of PI3K‐, AKT‐ or ERK pathways 205, which in turn limits the efficacy of mTORC‐inhibitors as targeted therapies 206. More recently developed dual ATP‐competitive agents that target mTORC1/mTORC2 have shown favourable results 207, 208 with AZD2014 effectively inhibiting PDAC cell division (G1 arrest), proliferation, and invasion *in vitro* 158, 160 and prolonging survival in the KPC mouse model of PDAC 109, 208, 209. However there is still some debate as to whether blocking mTORC1/2 leads to the adaptive activation of the PI3K‐AKT pathway 209, and consequently whether multiple targeting of this network is required to effectively interfere with both branches of adaptive signalling and to elicit a durable therapeutic response.

The combination of Cyclin‐dependent Kinase (CDK) inhibitors with PI3K pathway inhibition has been shown to inhibit tumour growth and metastasis in a variety of cancers including PDAC 210, 211, with a need for molecular stratification into responsive subtypes 212. Furthermore, multitarget, unique formulations, including SM‐88, a combination of a tyrosine derivative (D,L‐alpha‐metyrosine), mTOR inhibitor (sirolimus), CYP3a4 inducer (phenytoin) and oxidative stress catalyst (methoxsalen), are showing encouraging efficacy in early stage trials, particularly in patients with advanced pancreatic cancer (Table 2) 213, who have frequently exhausted all options. There is also ample evidence supporting the combination of PI3K/AKT/mTOR inhibitors with tyrosine kinase inhibitors (TKIs). Cancers with active/overexpressed TKIs often display resistance to TKIs through PI3K signalling 214. In addition, targeting RAS/RAF/MEK/ERK pathway in combination with PI3K/AKT/mTOR inhibitors is another promising strategy because there is significant stimulatory crosstalk 214. Synergy has previously been shown between a MEK‐inhibitor and PI3K/mTOR inhibitor in a lung cancer model, where inhibition of MEK/ERK was shown to stabilise BIM, and PI3K/AKT inhibition upregulated PUMA via FOXO, all of which are key mediators of apoptosis 215, 216. Inhibition of the MAPK pathway has also been shown to associate with increased PI3K pathway activity 217, 218. This therapeutic combination could also be beneficial in PDAC, as an alternative approach for inhibiting oncogenic *Kras*, which is located upstream of MEK/ERK and PI3K. Thus far, attempts at targeting the most frequently mutated protein in PDAC, KRAS, have been unsuccessful 14, 219. Whilst the combination of MEK inhibitors with alternative pathway inhibitors such as PI3K or Src has shown early promise 218, 220, 221, the combinations, including addition of chemotherapies, may require an alternative, intermittent dosing regimen design due to issues with chronic administration 222, 223, 224, and are yet to be systematically examined in PDAC. Preclinical data suggest that therapeutic efficacy may be dependent on PDAC subtype, as well as MEK activity and expression 225, with further investigation, including determination of biologically effective dose(s) of targeted therapies, testing and implementation of alternative dosing regimens, warranted.

Given the importance of the integrin/Src/FAK signalling in diverse cancer types, significant research has also gone into targeting molecules upstream of Src, including integrins, which critically modulates ECM mechanics and cytoskeleton stability, stellate cell activation 226, cancer cell survival and angiogenesis 59 and most recently, production of tumour‐promoting cytokines and chemokines 227. With each integrin comprising an α and β transmembrane subunit, most studies have focused on testing avβ1, avβ3, avβ5 integrin antagonists, the most promising of which is cilengitide. Cilengitide is an RGD (arginine‐glycine‐aspartic acid) peptide which is selective against avβ3, avβ5 integrins 228. Cilengitide was shown to have antitumour activity in recurrent and newly diagnosed glioblastoma 229, 230, 231, 232; however, further phase III studies showed no significant differences in median overall survival 231, with similar negative findings in PDAC when examined in all‐comers 233. In contrast, results from a phase I study suggest promising early signals of activity with cilengitide and chemoradiotherapy combination in advanced nonsmall cell lung cancer 234. Clinical trials of further integrin antagonists, including intetumumab, volociximab, ATN‐161 (Ac‐PHSCN‐NH2 peptide), abituzumab and etaracizumab, all of which are antibodies or peptide mimetics, have largely yielded no improvements in patient progression‐free or overall survival (Table 2) 235, 236; however, specific studies in colon cancer suggest that their antitumour activity may be linked to the presence of a biomarker 237, and, alternatively, may specifically inhibit the progression of bone‐associated metastases in prostate cancer 238. Adding to the complexity, anti‐integrin compounds may increase intratumoural hypoxia, leading to increased tumour growth, metastasis and chemoresistance in certain settings 239, 240, process that is dose‐ and/or tumour type‐dependent 65, 241. Reynolds *et al*. 241 showed that in fact, low (nanomolar) concentrations of avβ3, avβ5 inhibitors can paradoxically promote VEGF‐mediated angiogenesis by altering avβ3 integrin and VEGFR‐2 trafficking, stimulating cancer growth.

Hence, more recent research efforts have focussed on utilising these agents as part of ‘vascular normalisation’, whereby improved tumour blood flow increases drug delivery 242. However as this approach is highly time‐ and dose‐dependent, its clinical implementation may be challenging 243. Specifically, in pancreatic cancer, cilengitide has been effectively applied in combination with chemotherapy using a strategy called ‘vascular promotion’, aimed at improving delivery of chemotherapy to the tumour 244. Although the combination has yet to be trialled in the clinic, preclinical evidence is positive. Co‐administration of low‐dose therapy regimen of cilengitide and verapamil increased tumour blood flow and perfusion, promoted gemcitabine delivery inside growing pancreatic tumours, ultimately leading to reduced primary tumour growth, metastasis and significantly improved survival in multiple models of PDAC with minimal side effects 244. This dual therapy also increased levels of proteins involved in active transport of gemcitabine into cells, and production of active metabolites, further enhancing gemcitabine potency. Vascular promotion is also associated with reduced hypoxia and desmoplasia, salient features of PDAC 244. In addition, volociximab, an integrin α5β1 blocking antibody, has completed phase II trials in combination with gemcitabine in metastatic pancreatic cancer, with results pending (NCT00401570). Of note, mutant P53 has been shown to regulate α5β1 signalling and EGFR, which suggests there may also be potential for molecular stratification 245.

Another major advance in ECM‐targeting is the development of agents that break down hyaluronic acid (HA). HA is a large, linear, glycosaminoglycan that plays an important structural role in the ECM, and accumulates in conditions involving rapid and invasive cell division, including cancer. HA regulates interstitial gel fluid pressure within tumours, often impacting on drug delivery. Pegylated recombinant human hyaluronidase (PEGPH20) and 4‐methylumbelliferone are two key examples of compounds that inhibit and/or break down HA. Of note, PEGPH20 has already shown significant promise in PDAC. HA degradation following PEGPH20 treatment has been shown to normalise interstitial fluid pressures and re‐expand the microvasculature by increasing the diameter but not the total number of blood vessels within PDAC tumours 246. This in turn significantly improved chemotherapeutic response in the KPC murine model of PDAC, resulting in a near doubling of overall survival 246, 247. Clinical studies of PEGPH20 are also promising with phase II data already demonstrating significant efficacy of this agent when combined with chemotherapy, effect particularly prominent in patients with HA‐high tumours 248, highlighting the potential utility of intratumoural HA as a predictive biomarker of response 248, 249, 250. Favourable results are particularly observed when PEGPH20 is combined with Gemcitabine and Abraxane 248, 251, 252, whereas FOLFIRINOX in contrast may be better utilised in other settings 253. Development of a liquid biopsy‐based companion diagnostic for selecting potential PEGPH20 responders is also underway 254. Consequently several phase II/III clinical trials are now investigating further the clinical efficacy of PEGPH20, in combination with standard of care chemotherapies (Table 2) (NCT02487277, NCT02715804), or immune checkpoint inhibition (NCT03481920; NCT03634332, NCT03193190) in HA‐high molecular subgroups of PDAC 248, 255. These encouraging early clinical findings highlight the potential of stromal components as viable therapeutic targets, supporting further clinical development of PEGPH20 as well as detailed exploration of new biomarker‐driven therapeutic combinations utilising this agent.

## Future perspectives for inhibition of Src signalling in pancreatic cancer

The extraordinary and constantly expanding understanding of the role of Src signalling in pancreatic cancer biology and treatment supports the foundation for the specific inhibition of this complex network in PDAC. However, the presumption that a single‐targeted therapy will improve survival in such an aggressive disease is unrealistic. Unfortunately, most targeted therapies are at best only transiently effective, with cancer cells rapidly acquiring resistance, often leading to more rapid disease progression. This is supported by the numerous unsuccessful nonbiomarker‐driven clinical trials that have been summarised in this review.

Further understanding of the intricacies in integrin/Src/FAK and downstream signalling in the various tumour compartments will determine whether the inhibitors of this complex network may serve as effective treatments for newly diagnosed or recurrent tumours and will establish optimal combinations with radiation, cytotoxic chemotherapy and other targeted molecular compounds. Given the need for co‐targeting of multiple cancer capabilities to overcome the high therapeutic resistance of pancreatic tumours, future clinical applications of multiagent therapies will likely require a more innovative approach to dosing, including use of biologically effective doses of targeted agents (integrin/Src/FAK), and alternative dosing schedules such as ‘priming’ or ‘maintenance therapy’ to ensure maximal benefit to the patient 152. Finally, the emerging efficacy of Src pathway inhibitors in combination with other targeted and/or cytotoxic therapies, when examined in a molecular subtype‐specific context 248, 249, and with longitudinal tracking of long‐term therapeutic responsiveness, reveals significant potential as a personalised medicine strategy for pancreatic cancer, and provides real hope for patients in the future.

## Conflict of interest

The authors declare no conflict of interest.
